# Targeting the AGE-RAGE/NF-κB Pathway: An Integrative Study Decoding the Anti-Colitis Effect of Five-Flavor Sophora Flavescens Enteric-Coated Capsule

**DOI:** 10.3390/biomedicines14061236

**Published:** 2026-05-29

**Authors:** Baihui Hu, Xiaocong Wang, Xiuli Chen, Tong Li, Chuqiao Li, Yapu Zhang, Yingde Wang, Jingwei Mao

**Affiliations:** Department of Gastroenterology, Inflammatory Bowel Disease Center, The First Affiliated Hospital of Dalian Medical University, Dalian116000, China; hbaihui170@163.com (B.H.); wangcongcong981027@163.com (X.W.); kellyjade@163.com (X.C.); litong2024@dmu.edu.cn (T.L.); lcqjz1117@163.com (C.L.); zhangyapu123@163.com (Y.Z.)

**Keywords:** ulcerative colitis, Five-Flavor Sophora Flavescens Enteric-Coated Capsules, network pharmacology, integrative medicine, alleviate intestinal inflammation

## Abstract

**Background:** The Five-flavor Sophora Flavescens Enteric-coated Capsule (FSEC) is widely used to treat ulcerative colitis (UC), yet its underlying molecular mechanisms remain incompletely understood. This study aimed to systematically elucidate the therapeutic mechanisms and clinical value of FSEC by integrating network pharmacology, experimental validation, and clinical data. **Methods:** A multi-tiered methodological framework was implemented. First, network pharmacology analysis identified potential molecular targets and signaling pathways associated with FSEC in UC. These predictions, including the core targets and the AGE-RAGE/NF-κB signaling pathway, were subsequently validated in a dextran sulfate sodium-induced mouse colitis model in vivo and a lipopolysaccharide-stimulated RAW264.7 macrophage inflammation model in vitro. Finally, a single-center retrospective cohort study of 90 patients evaluated the efficacy and safety of FSEC as monotherapy and in combination with Mesalazine. **Results:** Network pharmacology identified 12 core targets and predicted the AGE-RAGE/NF-κB pathway as a key mechanism. Experimental validation demonstrated that FSEC inhibits this pathway. Both in vivo and in vitro studies consistently showed that FSEC downregulates RAGE expression and NF-κB p65 phosphorylation, reducing the production of key inflammatory mediators and thereby alleviating intestinal inflammation. Clinically, FSEC monotherapy achieved efficacy comparable to that of Mesalazine, and combination therapy was associated with higher clinical remission and mucosal healing rates, with a favorable safety profile. **Conclusions:** This comprehensive multi-omics investigation provides integrated evidence that FSEC exerts anti-inflammatory effects, associated with inhibition of the AGE-RAGE/NF-κB pathway. The observed synergy between FSEC and Mesalazine suggests the potential of an integrated therapeutic approach for UC.

## 1. Introduction

Ulcerative colitis (UC) is a chronic, idiopathic inflammatory disease of the colon and rectum, characterized by recurrent episodes of diarrhea, abdominal pain, anemia, and weight loss. The global incidence of UC has been increasing [1,2]. In China, the incidence has risen markedly in recent years, reaching 8.95 per 100,000 in 2016 [3]. Given its chronic, relapsing course and unclear etiology, it requires lifelong pharmacological management [4,5]. The main therapeutic agents include Mesalazine, corticosteroids, immunosuppressants, and biologics [6]. Although approximately 50% of patients achieve symptom relief with these treatments, their use is often limited by adverse events and the risk of drug dependence [7].

The Five-flavor Sophora Flavescens Enteric-coated Capsule (FSEC), a traditional Chinese herbal preparation composed of *Sophora flavescens*, *Radix sanguisorbae*, *Indigo naturalis*, *Bletilla striata*, and *licorice* [8], has been endorsed in several Chinese guidelines for the management of dampness-heat-type UC [9,10] and has shown favorable clinical application prospects. However, the pharmacological basis underlying its therapeutic effects remains incompletely defined. Although recent studies have begun to characterize its downstream pharmacological actions [11,12,13], the key upstream pathway orchestrating these effects remains unidentified. In particular, whether FSEC modulates the Advanced glycation end products-Receptor of AGE/Nuclear factor-kappa B (AGE-RAGE/NF-κB) signaling pathway—a central upstream regulator of inflammatory cascades—has not been experimentally investigated. 

The persistent and refractory inflammatory microenvironment in the colon poses a significant challenge in UC treatment. Sustained inflammation compromises the epithelial barrier, leading to increased permeability and facilitating the translocation of foreign antigens, which in turn perpetuates aberrant immune responses [14]. Simultaneously, activated macrophages release excessive pro-inflammatory cytokines and reactive oxygen species (ROS), exacerbating oxidative stress and tissue damage [15]. Traditional Chinese medicine (TCM) formulations, with their multi-target and holistic regulatory properties, have shown considerable potential in modulating UC-associated inflammation. For instance, Total Glucosides of Paeony (TGP) mitigate intestinal inflammation by inhibiting the release of inflammatory cytokines [16], while Qingchang Wenzhong Decoction improves colitis by restoring the balance between T helper 17 (Th17) and regulatory T (Treg) cells [17]. Recent advances in systems biology and the expanding availability of biomedical databases offer a novel paradigm for elucidating the polypharmacology of TCM formulations [18]. This study combines network pharmacology with experimental validation using a dextran sulfate sodium (DSS)-induced colitis mouse model and lipopolysaccharide (LPS)-stimulated RAW264.7 macrophages. The objective is to ascertain whether the anti-UC effects of FSEC are mediated through the AGE-RAGE/NF-κB signaling pathway. Additionally, a retrospective clinical cohort was analyzed to evaluate the clinical efficacy and safety of FSEC as monotherapy and in combination with Mesalazine. By linking computational prediction with experimental and clinical evidence, this work provides a foundation for understanding the molecular basis of FSEC and informing its rational clinical use. 

## 2. Material and Methods

### 2.1. Reagents

Details regarding all major drugs and reagents are available in Supplementary file S1 Table S1.

### 2.2. Network Pharmacology Analysis

The bioactive constituents of FSEC and their respective molecular targets were systematically retrieved from the Traditional Chinese Medicine Systems Pharmacology Database and Analysis Platform (TCMSP; https://www.91tcmsp.com/#/home, accessed on 6 June 2025). Bioactive compounds were screened using widely accepted thresholds: oral bioavailability (OB) ≥ 30% and drug-likeness (DL) ≥ 0.18. For herbs yielding fewer than five compounds, all reported constituents were included to ensure comprehensive coverage.

The putative protein targets of these compounds were initially retrieved as UniProt IDs or gene symbols. To ensure consistency, all targets were subsequently standardized to official human gene symbols using the UniProt Knowledgebase (UniProtKB; https://www.uniprot.org/, accessed on 16 June 2025) [19], with the following filtering criteria: (1) only entries with “reviewed” (Swiss-Prot) status were retained; (2) only human (Homo sapiens) entries were included; (3) ambiguous or uncharacterized protein entries were excluded.

Genes associated with UC were identified from the Gene Expression Omnibus (GEO; https://www.ncbi.nlm.nih.gov/geo/, access on 18 June 2025) dataset GSE24287, which contains colonic biopsies from active UC patients and healthy controls. Differential expression analysis was conducted using the limma package in R (version 4.1.0), applying a significance threshold of |logFC| > 0.5 and an adjusted *p*-value (Benjamini–Hochberg FDR) < 0.05. 

Potential therapeutic targets of FSEC for UC were identified by intersecting drug-target genes with UC-associated genes. The significance of this overlap was assessed using a one-tailed hypergeometric test, with all human protein-coding genes (*n* = 19,000) as the background. The overlapping gene set obtained was subsequently imported into the STRING database (https://stringdb.org/, version 11.5, accessed on 23 June 2025) to construct a protein–protein interaction (PPI) network. The following parameters were applied: interaction confidence score threshold ≥ 0.4 (medium confidence); maximum number of interactors set to 0 (no limit); active interaction sources limited to “experiments” and “databases” to reduce false positives. The resulting network was visualized using Cytoscape software (version 3.8.2) [20].

Core hub genes within this network were identified using the cytoHubba plugin in Cytoscape. The intersection of the top 15 genes from seven algorithms (MCC, MNC, Degree, Closeness, Betweenness, Stress, and EPC) constituted the core gene set. Gene Ontology (GO) functional annotation and Kyoto Encyclopedia of Genes and Genomes (KEGG) enrichment analyses were performed on this core set using clusterProfiler (version 4.0.5). This significance threshold was set at an adjusted *p*-value (Benjamini–Hochberg FDR) < 0.05 and q-value < 0.05. For mechanistic discussion, priority was given to KEGG pathways with an adjusted *p*-value < 0.001 and containing at least three core genes.

### 2.3. Animals and Experimental Design

Female C57BL/6 mice (6 weeks, 20–22 g) were procured from Liaoning Changsheng Biotechnology Co., Ltd. (License No. SCXK(Liao)2025-0001, Benxi, China). All mice were housed under specific pathogen-free (SPF) conditions at 20–24 °C and 40–60% humidity, with a 12 h light/dark cycle and ad libitum access to food and water. All procedures were conducted in accordance with the institution’s guidelines for the care and use of laboratory animals.

All C57BL/6 mice were randomly assigned to four groups (*n* = 5 per group): control (CON), the DSS-induced colitis model (DSS), the Mesalazine Enteric-Coated Tablets (MECT) and FSEC treatment. Acute colitis was induced by administering 3% DSS in drinking water for 7 days to all groups except the CON group, which received distilled water. From day 8 to day 14, mice in the MECT and FSEC groups received a daily enema containing MECT solution (0.5 g/kg/day) or FSEC solution (3.84 g/kg/day), respectively, each dissolved in 0.9% sodium chloride to a final volume of 0.2 mL. The CON and DSS groups received an equivalent volume of 0.9% sodium chloride via enema. For enema administration, a sterile tube was gently inserted 2 cm into the rectum to slowly instill the solution. The mouse was held in a head-down position for 30 s afterward to minimize fluid reflux. The enema dose was determined using the body surface area (BSA)-based dose conversion formula for converting clinical adult daily doses to mouse equivalents. The rectal route was selected to achieve high local drug concentrations in the colon, consistent with the pH-dependent release profile of the enteric-coated formulations. Compared with oral administration, this route reduces experimental variability caused by gastric emptying and upper gastrointestinal absorption and more directly models the clinical use of enema therapy in patients with distal UC.

Body weight, stool consistency, and fecal occult blood were recorded daily throughout the treatment period to evaluate the disease activity index (DAI) [21] (Supplementary file S1 Table S2). On day 14, after a 24 h fast with free access to water, all mice were sacrificed. Orbital blood samples were collected, centrifuged and stored at −80 °C for subsequent enzyme-linked immunosorbent assay (ELISA) analysis. After measuring the total length of the colon, the distal third of each colon was fixed in 4% paraformaldehyde for hematoxylin and eosin (HE) staining and immunohistochemical (IHC) analysis. The remaining colon tissue was preserved at −80 °C for subsequent Western blot and quantitative real-time PCR (qRT-PCR) analyses.

### 2.4. ELISA

The concentrations of AGEs in serum, along with interleukin-1 beta (IL-1β), interleukin-6 (IL-6) and tumor necrosis factor-alpha (TNF-α) in both serum and colon tissues, were measured in murine models using commercial ELISA kits, adhering strictly to the protocols specified by the manufacturers.

### 2.5. HE Staining

Colon tissues were fixed in 4% paraformaldehyde for 24 h, dehydrated through a graded ethanol series, embedded in paraffin, and sectioned. The sections were deparaffinized, rehydrated, stained with hematoxylin and eosin, dehydrated, and mounted. Morphological changes were examined under a light microscope. Histopathological scoring of inflammatory cell infiltration and tissue damage was performed according to the criteria detailed in Supplementary file S1, Table S3 [22]. Representative images were captured at a magnification of 200×.

### 2.6. IHC Staining

IHC staining was performed to evaluate the expression of RAGE, NF-κB p65, and phosphorylated NF-κB p65 (p-p65). Paraffin-embedded colon sections were deparaffinized, rehydrated, and subjected to antigen retrieval. Endogenous peroxidase activity was quenched via incubation with 3% H_2_O_2_ for 10 min. After blocking with 5% bovine serum albumin (BSA) for 1 h at room temperature, the sections were incubated overnight at 4 °C with the following primary antibodies: rabbit monoclonal anti-RAGE (1:500, 16346-1-AP, PTG, Wuhan, China), rabbit monoclonal anti-NF-κB p65 (1:50, CY2329, ABWAYS, Shanghai, China), and rabbit monoclonal anti-phospho-NF-κB p65 (1:100, CY6372, ABWAYS, Shanghai, China). Following TBST washing, the sections were incubated with a horseradish peroxidase (HRP)-conjugated secondary antibody (1:500, SA00001-2, PTG, Wuhan, China) for 1 h at room temperature. This procedure was followed by chromogenic visualization using 3,3’-diaminobenzidine (DAB) and counterstaining. The sections were mounted and examined under a light microscope, with images captured at a magnification of ×200. Quantitative analysis of IHC was performed using ImageJ software (version 1.53t).

### 2.7. Western Blot

Colon tissue and cell samples were homogenized and lysed in RIPA lysis buffer (Radio-Immunoprecipitation Assay buffer) supplemented with protease and phosphatase inhibitors. Protein concentration was quantified using a bicinchoninic acid (BCA) assay. Equal amounts of protein were resolved by sodium dodecyl sulfate-polyacrylamide gel electrophoresis (SDS-PAGE) and transferred onto 0.22 µm polyvinylidene fluoride (PVDF) membranes. The membranes were blocked with 5% non-fat milk for 1 h at room temperature and then incubated overnight at 4 °C with the following primary antibodies: rabbit anti-RAGE (1:1000, ab216329, Abcam, Cambridge, UK), rabbit anti-NF-κB p65 (1:1000, #8242, CST, Danvers, MA, USA), rabbit anti-phospho-NF-κB p65 (1:1000, #3033, CST, Danvers, MA, USA), and HRP-conjugated recombinant anti-GAPDH (1:3000, ZB15004-100, Servicebio, Wuhan, China) as a loading control. After washing, the membranes were incubated with an HRP-conjugated secondary antibody (1:10,000, SA00001-2, PTG, Wuhan, China) for 1 h at room temperature. Immunoreactive bands were visualized using a gel imaging system, and band intensities were quantified with ImageJ software. The expression levels of RAGE, p65, and p-p65 were normalized to GAPDH.

### 2.8. qRT-PCR

Total RNA was isolated from colon tissue and cell samples using the TRIzol reagent (AG21102, AG, Changsha, China) and reverse-transcribed into complementary DNA (cDNA) with the Evo M-MLV Reverse Transcription Kit (AG11728, AG, Changsha, China). qRT-PCR was performed using the SYBR Green Premix Pro Taq HS qPCR Kit (AG11701, AG, Changsha, China) with primers for TNF-α, IL-1β, IL-6, and RAGE (Supplementary file S1, Table S4). GAPDH served as the internal control. Relative mRNA expression was calculated using the 2^−^ΔΔCt method.

### 2.9. Cell Culture and Viability Assay

RAW264.7 macrophages were maintained in MEM supplemented with 10% fetal bovine serum and 1% penicillin/streptomycin at 37 °C in a 5% CO_2_ incubator. For the viability assay, cells were seeded in 96-well plates at 1 × 10^4^ cells /well (100 μL/well) and incubate for 24 h. The cytotoxic effects of FSEC and D-ribose (D-Rib) at various concentrations were evaluated using the Cell Counting Kit-8 (CCK-8) according to the manufacturer’s protocol. Absorbance was measured at 450 nm, and cell viability was calculated for each concentration. For subsequent experiments, cells were seeded in 6-well plates at 6 × 10^5^ cells/well and incubated for 12 h, then exposed to 1 μg/mL LPS for 6 h, followed by co-treatment with D-Rib for an additional 6 h. Cells were then treated with 600 μg/mL FSEC for 24 h. After microscopic examination, the culture supernatant and cells were harvested for further analysis.

### 2.10. Nitric Oxide (NO) Assay

Culture supernatants were collected, and NO concentration was measured using a commercial assay kit according to the manufacturer’s instructions. Absorbance was read at 540 nm, and the NO concentration in each sample was determined from a standard curve.

### 2.11. Patient Grouping

A total of 90 patients with mild-to-moderate UC were collected in this single-center retrospective cohort study conducted at the First Affiliated Hospital of Dalian Medical University between January 2022 and December 2024. The criteria for inclusion were as follows: (1) age ≥ 18 years; (2) diagnosis of mild-to-moderate UC according to the 2023 Chinese National Clinical Practice Guideline on Diagnosis and Management of UC [9], with disease severity evaluated using the modified Truelove and Witts classification [23]; (3) concurrent diagnosis of dampness-heat-type UC based on the International Clinical Practice Guideline on the Use of Traditional Chinese Medicine for UC [10]. The following were the exclusion criteria: (1) significant dysfunction of vital organs; (2) pregnancy or lactation; (3) known hypersensitivity to MECT or FSEC; (4) concomitant use of medications that could influence study outcomes, including corticosteroids, immunosuppressants, and biological agents within 3 months prior to enrollment; (5) incomplete clinical or follow-up data.

Based on the actual therapeutic regimen received (i.e., treatment allocation was based on real-world clinical practice and patient records, not prospective randomization), eligible patients were divided into three groups: (1) FSEC group (30 patients), which received 4 capsules of FSEC (0.4 g per capsule) three times daily; (2) MECT group (30 patients), which received 4 tablets of MECT (0.5 g per tablet) twice daily; (3) CTFM group (30 patients), which received combination therapy with FSEC and MECT at the same dosages as described above. The treatment duration was 8 weeks for all three groups. All patients were followed-up weekly via telephone or outpatient visits to monitor treatment adherence and adverse events.

### 2.12. Clinical Data Collection and Analysis

The following data were retrospectively collected from electronic medical records: age, sex, duration of UC, body mass index (BMI), smoking history, extraintestinal manifestations, disease extent according to the Montreal classification [24], clinical course, and disease activity assessed using the Truelove and Witts classification (mild or moderate). Baseline and post-treatment laboratory parameters related to UC were assessed, including complete blood count (CBC) measured by a hematology analyzer, albumin (ALB) measured via the bromocresol green method, erythrocyte sedimentation rate (ESR) measured using the Westergren method, and C-reactive protein (CRP) measured via immunoturbidimetry. Colonoscopy was performed at baseline and after 8 weeks of treatment to evaluate mucosal healing. All colonoscopy procedures were performed by gastroenterologists with at least 5 years of experience in inflammatory bowel disease (IBD).

### 2.13. Clinical Efficacy Evaluation

Clinical remission was defined as a modified Mayo score < 3 with no individual sub-score > 1 [9]. Mucosal healing was defined as a Mayo endoscopic sub-score (Mayo-ES) of 0 [9]. To ensure assessment reliability, all colonoscopy images were independently reviewed by two blinded gastroenterologists not involved in patient treatment. Discrepancies between the two reviewers were resolved by a third senior gastroenterologist. Interobserver agreement was calculated using Cohen’s kappa coefficient. All adverse events during treatment were recorded, classified according to the Common Terminology Criteria for Adverse Events (CTCAE) version 5.0, and followed-up until resolution. 

### 2.14. Statistical Analysis

All statistical analyses were performed using SPSS software (version 26.0) and R software (version 4.2.1). Data are presented as mean ± standard deviation (SD) for normally distributed variables, median (interquartile range, IQR) for non-normally distributed variables, and count (percentage) for categorical variables. Prior to group comparisons, the normality of data distribution for all continuous variables was assessed using the Shapiro–Wilk test, and the homogeneity of variances was evaluated using Levene’s test. For unadjusted between-group comparisons, one-way ANOVA with Tukey’s post hoc test or the Kruskal–Wallis H test with Dunn’s post hoc test with Bonferroni correction was applied as appropriate; within-group comparisons were performed using paired *t*-tests or Wilcoxon signed-rank tests; and categorical variables were compared using chi-square or Fisher’s exact tests. To address potential indication bias, Propensity Score Matching (PSM) was performed using 1:1 nearest-neighbor matching (FSEC vs. MECT) and 1:2 matching (combination therapy vs. pooled monotherapy) with a caliper width of 0.2, adjusting for baseline covariates. Interobserver agreement for mucosal healing was evaluated using Cohen’s kappa coefficient. To further control for confounding, multivariate logistic regression was conducted to estimate odds ratios (OR) with 95% confidence intervals for clinical remission and mucosal healing. Retrospective power analysis was performed using GPower software (version 3.1.9.7) to assess the statistical power for detecting clinically meaningful differences. Detailed statistical methods are provided in Supplementary file S1. A *p*-value of <0.05 was considered statistically significant, except where otherwise specified for multiple comparison corrections.

## 3. Results

### 3.1. Network Pharmacology Analysis

#### 3.1.1. Identification of Putative Targets for FSEC and UC

**Figure 1:**
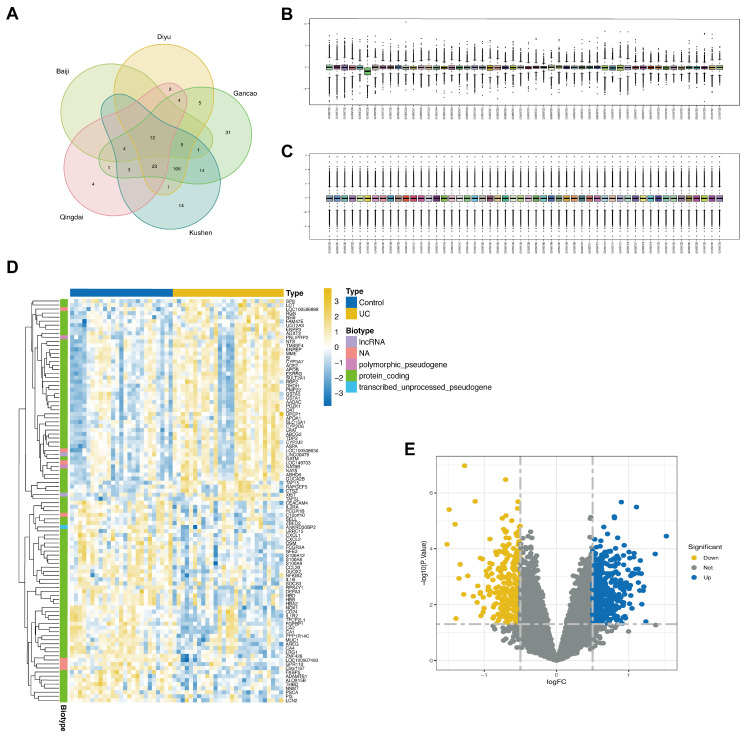
(**A**) Venn diagram of drug targets of five kinds of traditional Chinese medicine in FSEC. (**B**) The pre-normalization samples of GSE24287. (**C**) The post-normalization samples of GSE24287. (**D**) Heatmap of differential genes in UC, respectively, showing the top 50 upregulated and downregulated genes. (**E**) The volcano plot of differentially expressed genes in UC.

An investigation using the TCMSP database yielded 126 active compounds and 230 potential drug targets associated with the five herbal components of FSEC: *Sophora flavescens* (Kushen), *Radix sanguisorbae* (Diyu), *Indigo naturalis* (Qingdai), *Bletilla striata* (Baiji), and *licorice* (Gancao) (Supplementary file S2, Table S5). A Venn diagram of the putative targets of these herbs (Figure 1A) reveals overlapping targets, indicating that multiple herbal components may converge on common genes. The gene expression dataset GSE24287 was obtained from the GEO database to represent a cohort with UC. The distribution of raw, unnormalized sample data is illustrated in Figure 1B. Following the removal of batch effects (Figure 1C), differential expression analysis was conducted. A heatmap presents the top 50 upregulated and top 50 downregulated genes (Figure 1D), while a volcano plot illustrates all 682 DEGs (Figure 1E). The complete list of DEGs is provided in Supplementary file S2, Table S6.

#### 3.1.2. Identification of FSEC-UC Targets and Screening of Core Genes

**Figure 2:**
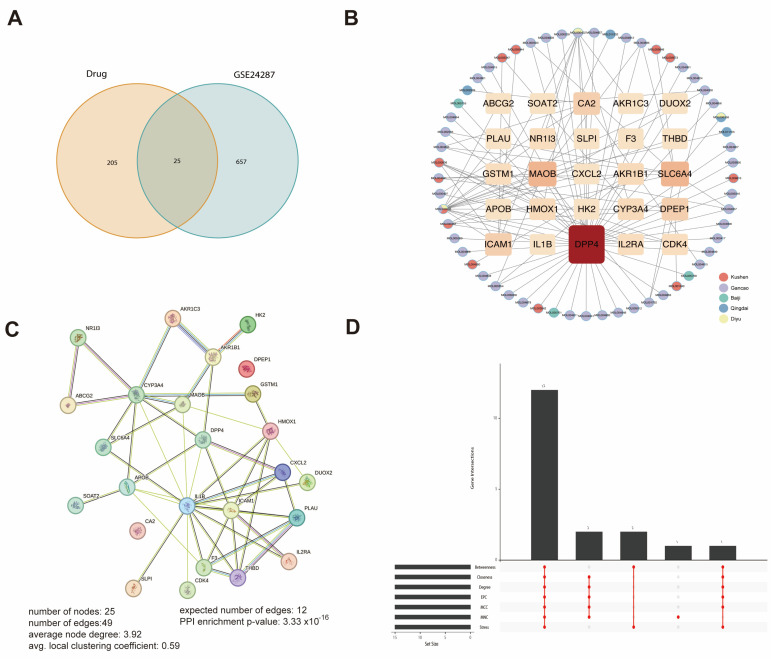
(**A**) The Venn diagram of drug genes and disease genes. (**B**) Network diagram of drug components and drug–disease targets. (**C**) The PPI network diagram of drug genes and disease genes. (**D**) Identification of 12 core genes by seven algorithms using a Venn diagram.

Intersection of the 230 FSEC-predicted targets with the 682 UC-associated DEGs yielded 25 overlapping genes (Figure 2A). Hypergeometric tests revealed that the 25 overlapping genes were more significant than expected by chance (*p* = 1.32 × 10^−5^), confirming a statistically significant enrichment of FSEC targets among UC-related genes. These overlapping genes were then integrated with the five active herbal components to construct a compound–target network in Cytoscape (Figure 2B). A PPI network was subsequently built using the STRING database (Figure 2C). The cytoHubba plugin was applied to identify the top 15 genes across seven distinct algorithms (detailed in Supplementary file S2, Table S7). Intersection of these results yielded 12 core genes consistently identified by all seven methods: IL1B, CYP3A4, APOB, DPP4, MAOB, ICAM1, HMOX1, F3, THBD, CXCL2, PLAU, and SLC6A4 (Figure 2D).

#### 3.1.3. GO Analysis and KEGG Pathway Enrichment Analysis

**Figure 3:**
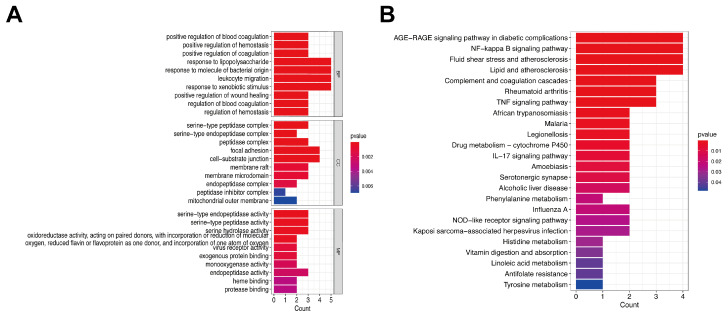
(**A**) GO analysis of core genes. (**B**) KEGG analysis of core genes. GO = Gene Ontology, KEGG = Kyoto Encyclopedia of Genes and Genomes.

To explore the therapeutic mechanisms of FSEC in UC, GO and KEGG enrichment analyses were performed on the core target set. The GO analysis revealed that the potentials targets of FSEC were significantly associated with 539 biological process (BP) terms, 34 cellular component (CC) terms, and 50 molecular function (MF) terms (Supplementary file S2, Table S8 and Figure 3A). Among these, FSEC predominantly influenced biological processes, such as positive regulation of hemostasis and coagulation, response to lipopolysaccharide, and response to bacterial-origin molecules. It is also involved in various molecular functions, including serine hydrolase/serine-type endopeptidase activity and oxidoreductase activity.

KEGG pathway analysis further identified several significant enriched pathways (Supplementary file S2, Table S9 and Figure 3B). The AGE-RAGE signaling pathway in diabetic complications (*p* = 7.40 × 10^−6^, gene count = 4) and the NF-κB signaling pathway (*p* = 8.99 × 10^−6^, gene count = 4) were among the most significantly enriched. Although multiple pathways met the significance threshold, the AGE-RAGE/NF-κB axis was selected for subsequent mechanistic validation based on the following considerations: (i) its recognized role as a master regulator of intestinal inflammation and epithelial barrier dysfunction in UC [25,26]; (ii) the presence of multiple core targets (e.g., IL1B, ICAM1) within this cascade; and (iii) its potential function as an upstream hub that integrates oxidative stress, immune activation, and cytokine production. Consequently, the AGE-RAGE and NF-κB signaling pathways have been recognized as critical mechanisms through which FSEC mediates its anti-UC effects. 

### 3.2. Therapeutic Effects of FSEC on UC in Mice

#### 3.2.1. FSEC Ameliorates DSS-Induced Colitis in Mice

**Figure 4:**
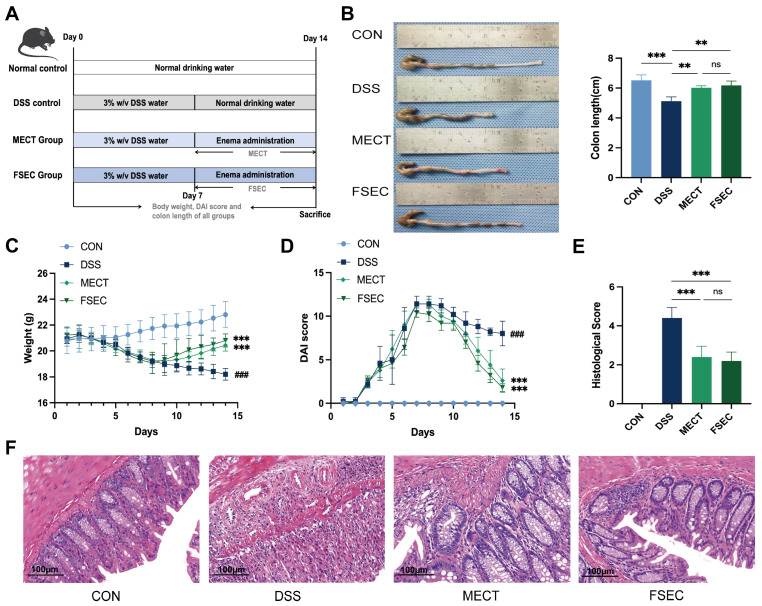
FSEC alleviates DSS-induced colitis in mice. (**A**) Schematic diagram of animal disease model establishment. (**B**) Changes in colon length, *n* = 5. (**C**) The time-course of body weight changes, *n* = 5. (**D**) DAI scores, *n* = 5. (**E**) Histological colonic score, *n* = 5. (**F**) Histological analysis of colon tissues by HE staining (×200, scale bars = 100 μm). Data are presented as mean ± SD. ^###^ *p* < 0.001 versus con groups, *** *p* < 0.001 versus DSS group, *** *p* < 0.001, ** *p* < 0.01. ns, no statistical significance.

Acute colitis was induced by 3% DSS administration to evaluate the protective effects of FSEC (Figure 4A). DSS-treated mice developed typical symptoms, including body weight loss, diarrhea, and hematochezia. Both MECT and FSEC treatment alleviated DSS-induced colon shortening (Figure 4B) and body weight loss (Figure 4C). Additionally, these treatments substantially reduced the DAI score (Figure 4D) and the histopathological score (Figure 4E). Collectively, these results indicate that both the MECT and FSEC interventions attenuated clinical symptoms and improved colonic histopathological damage in DSS-induced colitis.

#### 3.2.2. FSEC Attenuates Cytokine Expression in the Colon and Serum of DSS-Induced Mice

**Figure 5:**
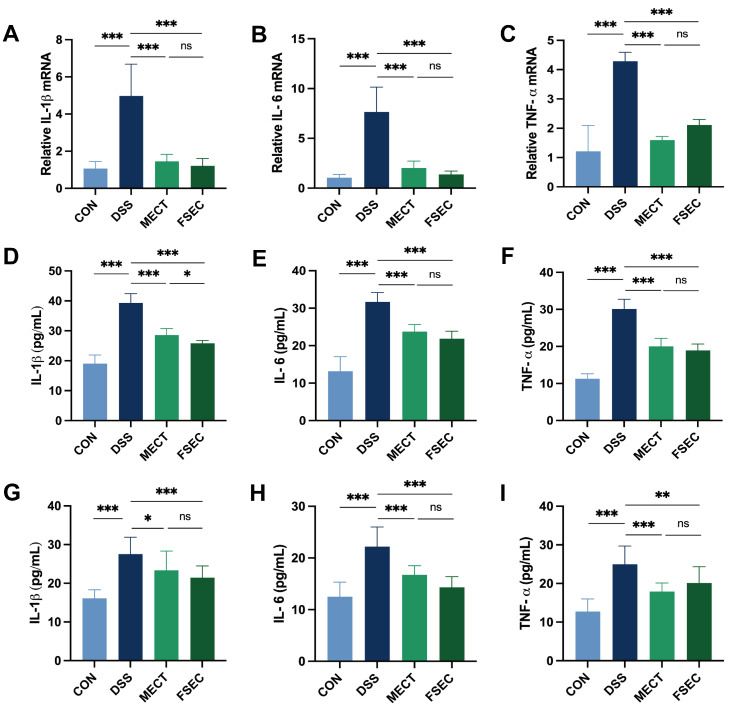
FSEC reduces the expression of inflammatory factors in the colon and serum of DSS-induced mice. (**A**–**C**) The mRNA expression levels of IL-1β, IL-6 and TNF-α in colonic tissue were measured by RT-qPCR, *n* = 3. (**D**–**F**) The concentrations of IL-1β, IL-6 and TNF-α in colon tissue were detected by ELISA, *n* = 5. (**G**–**I**) Serum concentrations of IL-1β, IL-6 and TNF-α were measured by ELISA, *n* = 5. Data are presented as mean ± SD. *** *p* < 0.001, ** *p* < 0.01, * *p* < 0.05. ns, no statistical significance.

IL-1β, IL-6, and TNF-α are pro-inflammatory cytokines implicated in UC pathogenesis. To assess the anti-inflammatory effects of FSEC at the molecular level, we quantified the mRNA expression of these cytokines in colonic tissue via qRT-PCR and measured their protein levels in colon tissues and serum using ELISA. DSS induction significantly elevated the mRNA expression of IL-1β, IL-6, and TNF-α in colonic tissue and increased their protein levels in both colon tissues and serum (Figure 5). In comparison to the DSS group, both FSEC and MECT treatment significantly reduced the colonic mRNA levels of these cytokines, and ELISA results confirmed a corresponding decrease at the protein level. These findings indicate that FSEC suppresses pro-inflammatory cytokine production at both local colonic and systemic levels.

#### 3.2.3. FSEC Attenuates UC-Induced Inflammation via Modulation of the AGE-RAGE/NF-κB Signaling Pathway

**Figure 6:**
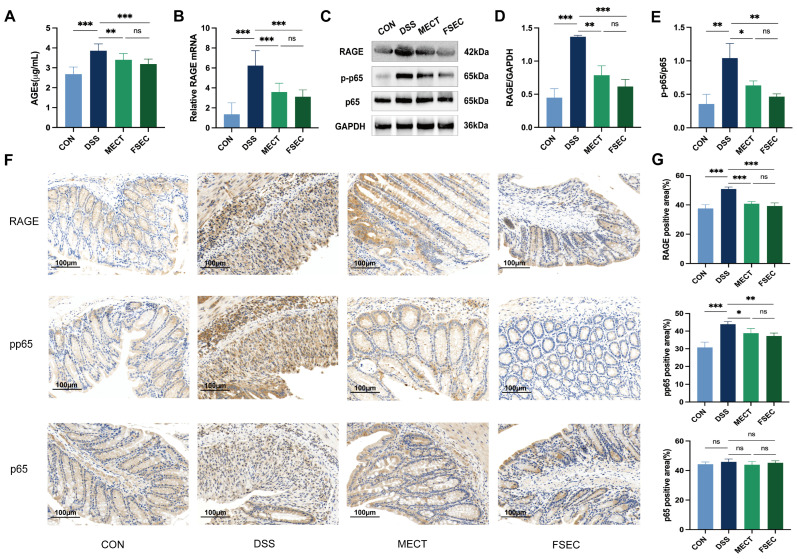
FSEC may inhibit the inflammation of UC by regulating the AGE-RAGE/NF-kB signaling pathway. (**A**) Serum concentrations of AGEs were measured by ELISA, *n* = 5. (**B**) The mRNA expression levels of RAGE in colonic tissue were measured by RT-qPCR, *n* = 3. (**C**) The protein expression levels of RAGE (**D**), p-p65 and p65 (**E**) in the colon tissue, *n* = 3. (**F**) Representative immunohistochemistry staining images of RAGE, p-p65 and p65 of colon tissues in each group, *n* = 5 (×200, scale bars = 100 μm). (**G**) Quantitative analysis of the percentage of positive staining area for RAGE, p-p65 and pp65. *** *p* < 0.001, ** *p* < 0.01, * *p* < 0.05. ns, no statistical significance.

To determine whether the anti-inflammatory effects of FSEC involve the AGE-RAGE/NF-κB pathway, we assessed key molecular markers via qRT-PCR, ELISA, Western blot, and IHC (Figure 6). DSS induction markedly elevated serum AGE levels, upregulated RAGE mRNA and protein expression in colonic tissue, and enhanced RAGE immunostaining in the colonic epithelium compared with the CON group. It also increased the p-p65 protein level, raised the p-p65/p65 ratio, and enhanced p-p65 nuclear translocation, without affecting total p65 levels. FSEC treatment reversed these changes by reducing serum AGE levels, downregulating RAGE expression, and inhibiting p-p65 upregulation and nuclear translocation. These results suggest that the anti-inflammatory properties of FSEC in UC are associated with modulation of the AGE-RAGE/NF-κB signaling pathway.

### 3.3. FSEC Ameliorates Macrophage Inflammation via the AGE-RAGE/NF-κB Pathway

**Figure 7:**
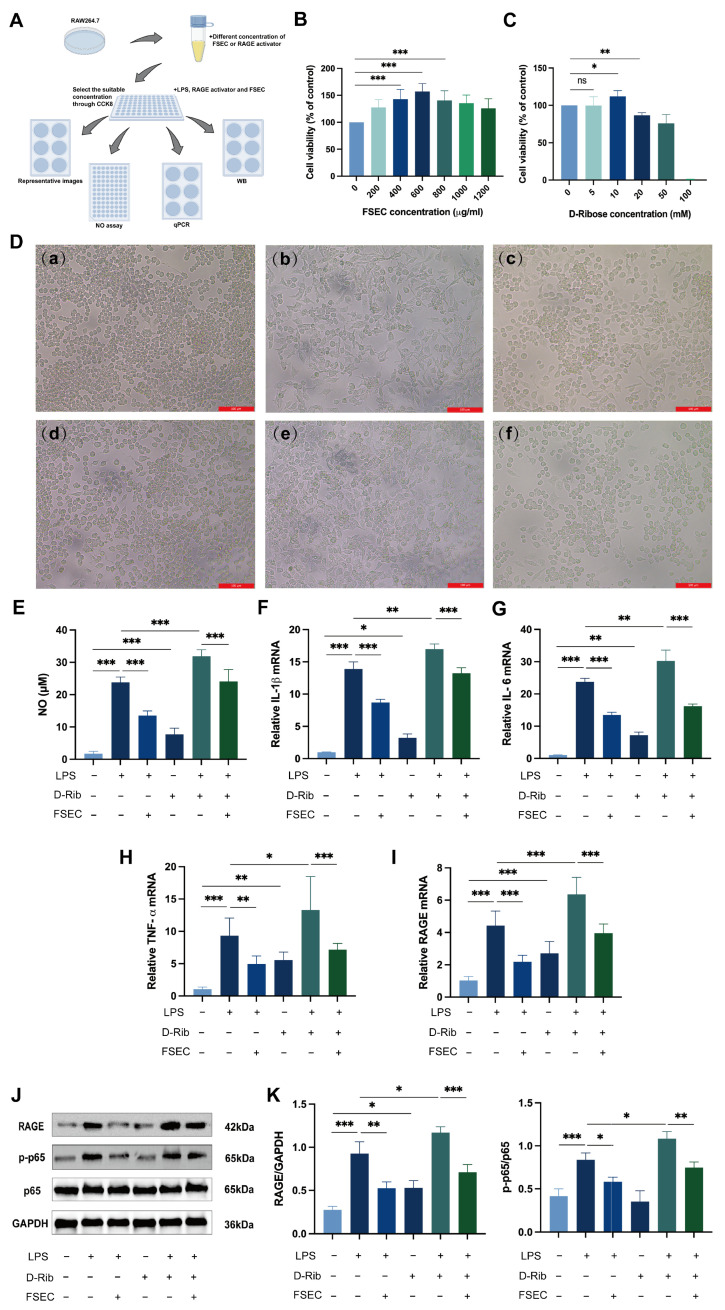
FSEC inhibits LPS-induced inflammation in RAW264.7 cells via regulating the AGE-RAGE/NF-κB signaling pathway. (**A**) Procedure for the in vitro study. (**B**,**C**) CCK-8 assay for cell viability following treatment with different concentrations of FSEC (**B**) or D-Ribose (**C**). (**D**) Representative images following various treatments ((**a**): Negative control group, (**b**): LPS group, (**c**): LPS + FSEC group, (**d**): D-Rib group, (**e**): LPS + D-Rib group, (**f**): LPS + D-Rib + FSEC group) (×200, scale bars = 100 μm). LPS was used at 1 μg/mL, D-Ribose at 10 mM, and FSEC at 600 μg/mL. (**E**) NO production in the culture supernatant measured by Griess assay. (**F**–**I**) The IL-1β, IL-6, TNF-α, and RAGE mRNA expression by qPCR. (**J**,**K**) Western blot and semi-quantification. Data are presented as mean ± SD. *** *p* < 0.001, ** *p* < 0.01, * *p* < 0.05. ns, no statistical significance.

To explore the cellular anti-inflammatory mechanism of FSEC, we initially assessed its cytotoxicity with the CCK-8 assay. FSEC enhanced RAW264.7 cell viability, and 600 μg/mL was selected for subsequent experiments (Figure 7B). D-Rib, a RAGE agonist, showed a biphasic effect on cell viability; a concentration of 10 mM was chosen as both safe and moderately active (Figure 7C). FSEC treatment reversed the LPS-induced morphological changes in RAW264.7 cells, including cell enlargement and increased pseudopodia formation. This protective effect was also evident under the more severe inflammation conditions induced by co-stimulation with LPS and D-Rib (Figure 7D). In established in vitro inflammation models, FSEC significantly reduced excessive NO production (Figure 7E) and downregulated the mRNA expression of IL-1β, IL-6, and TNF-α (Figure 7F–H). Mechanistically, RAGE expression was upregulated by LPS and further amplified by D-Rib, whereas FSEC effectively suppressed this upregulation at both the mRNA and protein levels (Figure 7I–K). Accordingly, FSEC inhibited the phosphorylation of NF-κB p65, with a more pronounced effect observed in the LPS plus D-Rib model (Figure 7J,K). These findings indicate that FSEC attenuates macrophage inflammation by inhibiting the AGE-RAGE/NF-κB signaling pathway.

### 3.4. Efficacy and Safety of FSEC in the Treatment of Mild-to-Moderate UC

#### 3.4.1. Baseline Characteristics

Based on the predefined inclusion and exclusion criteria, a total of 90 patients with mild-to-moderate UC were recruited. Baseline demographic and clinical characteristics did not differ significantly among the three treatment groups (all *p* > 0.05), ensuring the validity of subsequent efficacy comparisons. The detailed baseline characteristics of the patients are described in Table 1.

PSM was performed to balance baseline covariates. In the 1:1 PSM analysis, all 30 patients in the FSEC group were successfully matched with 30 patients in the MECT group. In the 1:2 PSM analysis, all 30 patients in the CTFM group were matched with 60 patients from the pooled monotherapy cohort. After matching, all standardized mean differences (SMDs) were below 0.1 (Supplementary file S3, Tables S10 and S11), confirming that PSM effectively controlled for potential confounding.

#### 3.4.2. Clinical Remission Rates and Mucosal Healing Rates

**Figure 8:**
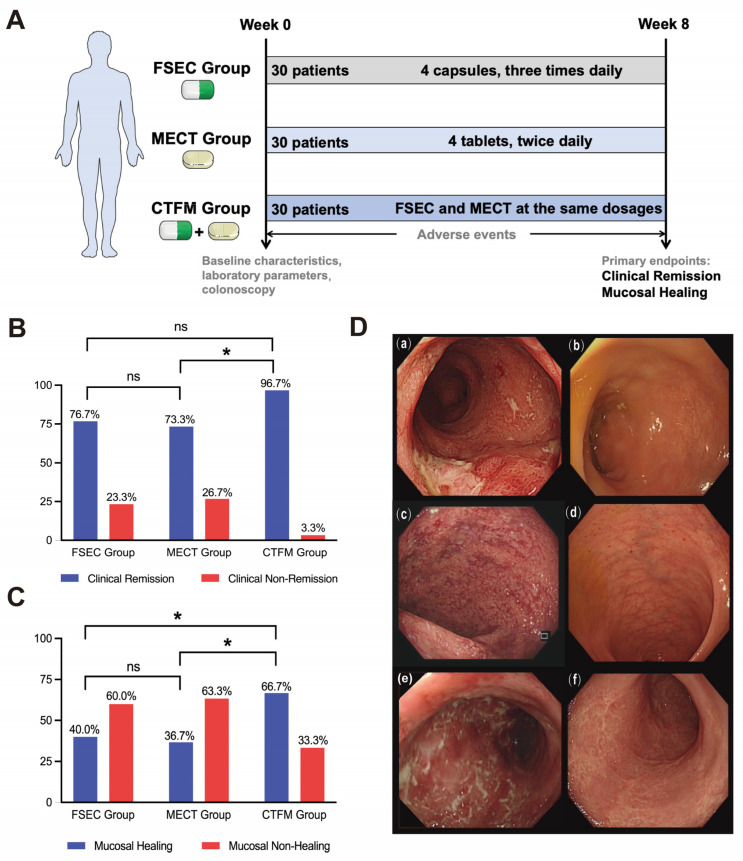
FSEC improves clinical outcomes in patients with UC. (**A**) Schematic diagram of the single-center retrospective cohort study design. (**B**,**C**) Comparison of clinical remission rate (**B**) and mucosal healing rate (**C**) among the three groups after 8 weeks of treatment. Data are presented as percentages of patients. * Corrected *p* < 0.0167 (Bonferroni correction); (**D**) Representative colonoscopy images from the FSEC group before (**a**,**c**,**e**) and after (**b**,**d**,**f**) treatment, showing substantial mucosal healing. These images are provided for illustrative purposes only to demonstrate the morphological changes associated with mucosal healing and do not constitute statistical evidence for the study conclusions. * *p* < 0.05. ns, no statistical significance.

The clinical remission rate in the CTFM group (96.7%) was higher than that in the FSEC group (76.7%) and the MECT group (73.3%). After Bonferroni correction for multiple comparisons, the difference between the CTFM group and the MECT group remained statistically significant (corrected *p* = 0.015), while the difference between the CTFM group and the FSEC group approached statistical significance (corrected *p* = 0.052). There was no statistically significant difference between the FSEC and MECT groups (χ^2^ = 0.089, *p* = 0.766) (Figure 8B).

The mucosal healing rate in the CTFM group (66.7%) was higher than that in the FSEC group (40.0%) and the MECT group (36.7%). After Bonferroni correction, the difference between the CTFM group and the MECT group remained statistically significant (corrected *p* = 0.012), and the difference between the CTFM group and the FSEC group was also statistically significant (corrected *p* = 0.031). There was no statistically significant difference between the FSEC and MECT groups (χ^2^ = 0.067, *p* = 0.796) (Figure 8C). 

#### 3.4.3. Interobserver Agreement Analysis

Interobserver agreement for mucosal healing assessment between the two independent reviewers was excellent, with a Cohen’s kappa coefficient of 0.87 (95% CI: 0.76–0.98). This indicates that the assessment of mucosal healing was highly reliable and not subject to significant observational bias.

#### 3.4.4. Statistical Robustness Analyses for Primary Comparisons

After PSM, the FSEC and MECT groups showed comparable rates of clinical remission (76.7% vs. 73.3%, χ^2^ = 0.089, *p* = 0.766) and mucosal healing (40.0% vs. 36.7%, χ^2^ = 0.067, *p* = 0.796), confirming that the comparable efficacy of the two monotherapies was not attributable to baseline imbalances. In contrast, the CTFM group achieved significantly higher rates of both clinical remission (96.7% vs. 71.7%, χ^2^ = 7.680, *p* = 0.006) and mucosal healing (66.7% vs. 36.7%, χ^2^ = 7.200, *p* = 0.007) compared with the matched monotherapy cohort, indicating that the superiority of combination therapy was robust to adjustment for baseline confounders.

Multivariate logistic regression analysis confirmed that treatment with CTFM was independently associated with higher odds of clinical remission (OR = 12.67, 95% CI: 1.52–105.63, *p* = 0.019) and mucosal healing (OR = 3.78, 95% CI: 1.32–10.82, *p* = 0.013) compared to pooled monotherapy. In contrast, there was no significant difference in the odds of clinical remission (OR = 1.18, 95% CI: 0.35–3.97, *p* = 0.789) or mucosal healing (OR = 1.14, 95% CI: 0.39–3.33, *p* = 0.812) between FSEC and MECT monotherapy. These results further validate the findings from the PSM analysis.

A retrospective power analysis for the comparison of clinical remission rates between FSEC and MECT monotherapy yielded 82.3% power to detect a 15% absolute difference (α = 0.05, two-sided), indicating that the study was adequately powered to detect a clinically meaningful difference. The non-significant result is, therefore, unlikely to be due to an insufficient sample size but rather reflects the true comparable efficacy of FSEC and MECT monotherapy.

#### 3.4.5. Comparison of Laboratory Parameters Related to UC at Baseline and After Treatment

In the FSEC group, CRP and ESR levels decreased after treatment compared with baseline, while PLT and ALB levels increased. In the MECT group, WBC, CRP, and ESR levels decreased after treatment compared with baseline, whereas the ALB level increased. In the CTFM group, CRP and ESR levels decreased after treatment compared with baseline, and the ALB level increased (Table 2 and Supplementary file S3, Tables S12–S14). It is important to note that these intra-group comparisons only demonstrate that each treatment regimen was effective in improving laboratory parameters, and they do not provide evidence for comparative efficacy between groups.

#### 3.4.6. Changes in Laboratory Parameters Related to UC at Baseline and After Treatment

Inter-group changes in laboratory parameters are summarized in Table 3. The reduction in the CRP level from baseline to post-treatment in the CTFM group was greater than that in the MECT group. However, no significant differences were observed between the FSEC and MECT groups or between the FSEC and CTFM groups. After Bonferroni correction for multiple comparisons, the difference in CRP reduction between the CTFM and MECT groups remained statistically significant (corrected *p* = 0.045).

#### 3.4.7. Adverse Events

The incidence of adverse events in the FSEC, MECT, and CTFM groups was 6.7%, 13.3%, and 6.7%, respectively, with no statistically significant differences among the groups (χ^2^ = 1.06, *p* = 0.72). All adverse events were classified as grade 1 or 2 according to CTCAE version 5.0, and no serious adverse events (grade 3 or higher) were observed in any group. Detailed data are presented in Supplementary file S3, Table S15.

In the FSEC group, one patient experienced mild nausea that resolved spontaneously without treatment, and one patient had a mild elevation in alanine aminotransferase (ALT) level (1.2-times the upper limit of normal) that returned to normal within 2 weeks after treatment completion. In the MECT group, one patient developed a mild skin rash, two experienced mild nausea, and one had mild dizziness. All these adverse events resolved spontaneously without pharmacological intervention. In the CTFM group, one patient developed a mild skin rash that improved after oral loratadine for 3 days, and one patient experienced mild nausea that resolved gradually without medication. No patients discontinued treatment due to adverse events.

## 4. Discussion

Within the framework of multidisciplinary systems biology, network pharmacology functions as a “Rosetta Stone” for unraveling the complex mechanisms of TCM by linking drug actions to biological targets and pathways [27]. Using this approach, we identified 12 core targets through which FSEC may act in UC. Among them, IL-1β—a potent macrophage-derived pro-inflammatory cytokine—functions as both an initiator and amplifier of the inflammatory response. Its expression is markedly upregulated in the colonic mucosa of UC patients and correlates positively with disease severity [28]. Activated NF-κB is a key transcription regulator of IL-1β, while IL-1β itself can further activate NF-κB, creating a positive feedback loop that perpetuates an inflammatory “storm”. Given this central role, we prioritized IL1β for experimental validation. Our experiments demonstrated that FSEC significantly reduced the expression of IL-1β, IL-6, and TNF-α in a DSS-induced colitis mouse model.

Our findings align with existing research on herbal formulations. Shaoyao decoction, for instance, reduces IL-1β levels through the MKP1/NF-κB pathway [29], and Jinmu-Tang mitigates UC inflammation by inhibiting inflammasome activation and decreasing IL-1β production [30]. Beyond IL-1β, the other predicted targets—spanning leukocyte adhesion (ICAM1) [31], neutrophil chemotaxis (CXCL2) [32], oxidative stress defense (HMOX1) [33], coagulation-fibrinolysis balance (F3, THBD, PLAU) [34,35,36], neuroimmune regulation (SLC6A4, MAOB) [37], and metabolic/mucosal repair (DPP4, CYP3A4, APOB) [38,39,40]—are detailed in Table 4 and were not directly measured in this study. Future studies incorporating broader target profiling are warranted. Collectively, these targets form a coordinated network of inflammation amplification, cell adhesion, oxidative stress, and immune regulation, reflecting the multi-target, holistic regulatory capacity of TCM and providing a systems biology framework for the principle of “clearing heat and dampness, promoting blood circulation, and detoxifying” in UC.

KEGG enrichment analysis identified the AGE-RAGE signaling pathway as a potential central mechanism for FSEC in UC. This pathway is a pivotal regulator of inflammation and oxidative stress in UC pathology. AGEs, formed through non-enzymatic glycation under hyperglycemic or oxidative conditions, bind to RAGE and trigger downstream inflammatory cascades [45]. AGE-RAGE engagement facilitates the recruitment of NF-κB p65 to the RAGE promoter, enhancing RAGE transcription and establishing a positive feedback loop that drives NF-κB p65 phosphorylation and nuclear translocation, ultimately leading to the expression of pro-inflammatory factors such as TNF-α and IL-6, which further exacerbate oxidative stress and inflammatory responses [46,47,48,49].

Based on this mechanism, we hypothesized that FSEC intervenes in UC through multi-target modulation. Our findings align with prior studies on herbal formulations targeting the AGE-RAGE axis in colitis models. Wumei Wan attenuates DSS-induced colitis and intestinal barrier damage by inhibiting RAGE expression and NF-κB activation [50]. Xiahuo Pingwei San reduces IL-6, IL-1β, and TNF-α levels in UC mice by suppressing the AGE-RAGE pathway [51]. In a rat model of type 2 diabetes with IBD), Ge-Gen-Qin-Lian Decoction alleviated intestinal symptoms and lowered blood glucose by blocking the AGE-RAGE pathway and downregulating c-JUN/NF-κB expression [26]. The involvement of this pathway in other inflammatory diseases, including diabetes, chronic atrophic gastritis, and cardiovascular diseases, further supports its relevance as a therapeutic target [52,53,54,55].

To test whether the anti-inflammatory effect of FSEC is functionally associated with the AGE-RAGE axis, we employed D-Ribose, a specific RAGE agonist, to selectively over-activate this pathway in RAW264.7 macrophages. The selective activation of RAGE by D-Ribose confirmed the centrality of this receptor in amplifying inflammatory signals, consistent with prior reports [56,57]. Despite this targeted over-activation, FSEC retained its inhibitory capacity against RAGE expression, NF-κB p65 phosphorylation, and downstream cytokine production, indicating that its anti-inflammatory action is functionally associated with the AGE-RAGE/NF-κB axis. This mode of action is consistent with that of other herbal formulations, such as the Sishen pill [58], although direct evidence establishing this pathway as the obligate mechanism of FSEC requires further investigation.

Prior studies have explored the anti-UC mechanisms of FSEC (also termed Composite Sophora Colon-Soluble Capsule, CSCC) from multiple perspectives. Early network pharmacology analyses identified quercetin, kaempferol, luteolin, and mangiferin as primary active constituents, with IL-17, TNF, Toll-like receptor, NF-κB, and Th17 cell differentiation predicted as key pathways [8]. Subsequent experimental work has provided mechanistic support for these predictions: CSCC attenuates DSS-induced colitis by restoring gut microbiota composition and modulating the Th17/Treg balance [11]; it promotes butyric acid production, which in turn regulates the ratio of NCR^+^ ILC3s to Lti ILC3s and enhances epithelial barrier function [12]; and it upregulates GPR43 to inhibit the MEK4/JNK1/STAT3 pathway, thereby suppressing NLRP3 inflammasome activation and IL-1β production [13]. Collectively, these studies underscore the multifaceted regulatory properties of FSEC, spanning gut microbiota modulation, epithelial barrier protection, and immune–inflammatory pathway inhibition. However, the upstream signaling hub through which FSEC orchestrates these diverse effects remains unclear. The present study addresses this gap by identifying the AGE-RAGE/NF-κB axis as a key upstream mechanism linking FSEC to its downstream anti-inflammatory actions.

Our clinical findings from this retrospective cohort indicate that FSEC monotherapy is associated with efficacy comparable to that of Mesalazine in mild-to-moderate UC, corroborating an independent clinical trial [59]. Combination therapy was associated with higher rates of clinical remission and mucosal healing, without additional safety concerns. These results were reinforced by several statistical approaches implemented to address the limitations of a retrospective design. PSM and multivariate logistic regression were applied to control for indication bias and baseline confounding, and both yielded results consistent with the unadjusted analyses. Excellent interobserver agreement ensured the reliability of mucosal healing assessment, and the benefits of combination therapy remained significant after Bonferroni correction. A retrospective power analysis confirmed that the study was adequately powered to detect a clinically meaningful difference between monotherapies, supporting the interpretation of genuinely comparable efficacy. All three regimens produced within-group improvements in clinical and laboratory parameters. However, inter-group comparison revealed that the reduction in CRP was significantly greater with CTFM than with MECT alone, a difference not observed in the other pairwise comparisons. This may reflect the multi-component, multi-target mechanisms of FSEC, which could exert a dominant anti-inflammatory effect within the combination regimen, although the limited sample size warrants cautious interpretation. Collectively, the data provide an analytical framework supporting the comparable effectiveness of FSEC and MECT as monotherapies, and they lend support to the potential for enhanced therapeutic benefits when FSEC is combined with conventional therapy.

This synergy points to a complementary mechanism between TCM and Western medicine. MECT, a classic aminosalicylate, exerts local anti-inflammatory effects in the intestine [60]. FSEC, by contrast, exemplifies the systemic, multi-target approach of TCM. According to the published literature, Kushen eases colitis by inhibiting the PI3K/AKT/mTOR pathway and reducing ferroptosis [61,62]. Diyu enhances macrophage function through antioxidant effects and autophagy induction [63,64]. Qingdai regulates the aryl hydrocarbon receptor (AHR), gut microbiota, and TLR4/MyD88/NF-κB pathway [65,66]. *Bletilla striata* polysaccharide (BSP) aids intestinal repair via coagulation activation and collagen cross-linking [67,68], and Gancao provides antioxidant and anti-inflammatory benefits through the Nrf2/PINK1 and NOD2/RIP2/NF-κB pathways [69,70]. Although these mechanisms were not directly verified in the present study, they offer a plausible framework for understanding how the components of FSEC may converge on the AGE-RAGE/NF-κB signaling hub. Thus, combining MECT with FSEC integrates rapid local anti-inflammatory effects with systemic, multi-target regulation, and may represent a promising strategy warranting further evaluation for long-term UC management.

Several limitations should be acknowledged. Firstly, the FSEC used in this study was not independently characterized by High-Performance Liquid Chromatography (HPLC) or mass spectrometry, and the network pharmacology analysis depended on public databases with inherent constraints, including incomplete compound libraries and reliance on a single UC dataset. Future studies integrating chemical fingerprinting with multi-source database analyses would improve both compound characterization and target prediction. Secondly, our data demonstrate that FSEC inhibits the AGE-RAGE/NF-κB pathway but do not establish a definitive causal link. RAGE knockout, NF-κB p65 silencing, or specific pharmacological blockade (e.g., FPS-ZM1) will be needed to determine whether FSEC acts obligately through this axis. Thirdly, this single-center retrospective cohort study has a modest sample size and lacks direct mechanistic evidence from patients. Although PSM and multivariate regression were used to control for observed confounders, unmeasured confounding may still influence the results. Future multi-center prospective trials incorporating pathway-specific biomarkers are needed to validate these findings clinically and explore their potential for predicting FSEC treatment response, thereby advancing precision treatment of UC with integrated traditional Chinese and Western medicine.

## 5. Conclusions

By integrating network pharmacology, experimental validation, and clinical data, this study demonstrates that FSEC treatment attenuates UC, and its effect is linked to the modulation of the AGE-RAGE/NF-κB signaling pathway. The clinical synergy observed with the FSEC–Mesalazine combination highlights a promising therapeutic strategy worthy of further investigation. These findings contribute to the mechanistic understanding of FSEC and support its further development as an integrated treatment option for UC.

## Figures and Tables

**Table 1:** Baseline characteristics of three groups of patients with mild-to-moderate UC.

Variables	FSEC Group (*n* = 30)	MECT Group (*n* = 30)	CTFM Group (*n* = 30)	*p* Value
Age (years)	47.23 ± 15.55	50.57 ± 16.33	54.07 ± 16.96	0.27
Gender-male (*n*)	17	15	20	0.42
Duration of UC (month)	24.00 (9.50, 44.50)	24.00 (1.00, 61.75)	27.00 (2.00, 93.75)	0.68
BMI	21.70 (19.58, 22.75)	21.85 (20.43, 23.65)	21.64 (20.40, 23.68)	0.82
Non-smoker (*n*)	23	29	25	0.08
No EIMs (*n*)	26	26	25	0.91
Disease extent (*n*)				0.07
E1	5	10	2	
E2	10	12	13	
E3	15	8	15	
Clinical course (*n*)				0.22
First episode	6	12	8	
Chronic continuous	24	18	22	
Severity (*n*)				0.18
Mild	14	16	8	
Moderate	16	14	21	

Abbreviations: UC, ulcerative colitis; FSEC, Five-Flavor Sophora Flavescens Enteric-coated Capsules; MECT, Mesalazine Enteric-Coated tablet; CTFM, combination therapy of FSEC and MECT; BMI, Body Mass Index; EIMs, extraintestinal manifestations.

**Table 2:** Laboratory parameters pre- and post-treatment in the three groups of patients.

Parameter	FSEC Group (*n* = 30)			MECT Group (*n* = 30)			CTFM Group (*n* = 30)		
	Pre	Post	*p*	Pre	Post	*p*	Pre	Post	*p*
WBC	6.17 ± 1.76	6.53 ± 2.77	0.49	6.46 ± 2.01	5.77 ± 1.35	<0.05	7.02 ± 2.65	6.51 ± 1.84	0.28
NE	3.77 ± 1.26	3.86 ± 2.11	0.81	3.62 ± 1.46	3.60 ± 0.96	0.95	4.45 ± 2.00	4.13 ± 1.75	0.27
RBC	4.36 ± 0.45	4.45 ± 0.38	0.34	4.47 ± 0.59	4.57 ± 0.53	0.31	4.25 ± 0.46	4.43 ± 0.63	0.10
HB	127.13 ± 15.73	128.63 ± 10.94	0.51	132.50 ± 17.99	135.43 ± 15.15	0.15	122.00 ± 16.82	126.10 ± 10.04	0.052
PLT	250.77 ± 54.73	283.07 ± 54.58	<0.05	241.86 ± 61.81	248.62 ± 58.73	0.94	265.70 ± 73.90	270.03 ± 67.18	0.71
CRP	8.14 ± 6.91	4.90 ± 5.15	<0.05	5.63 ± 4.78	3.77 ± 3.26	<0.05	21.89 ± 34.68	8.47 ± 12.58	<0.05
ESR	17.07 ± 16.61	8.60 ± 5.74	<0.05	15.62 ± 11.79	9.14 ± 7.94	<0.05	24.60 ± 24.85	15.76 ± 18.88	<0.05
ALB	39.31 ± 3.38	41.00 ± 2.16	<0.05	39.73 ± 3.48	41.65 ± 2.64	<0.05	36.62 ± 5.74	39.89 ± 3.55	<0.05

Abbreviations: FSEC, Five-Flavor Sophora Flavescens Enteric-Coated Capsules; MECT, Mesalazine Enteric-Coated Tablet; CTFM, combination therapy of FSEC and MECT; Pre, Pre-treatment; Post, Post-treatment; *p*, *p*-value; WBC (×10^9^/L), white blood cell count; NE (×10^9^/L), neutrophil count; RBC (×10^12^/L), red blood cell count; HB (g/L), hemoglobin; PLT (×10^9^/L), platelet count; CRP (mg/L), C-reactive protein; ESR (mm/h), erythrocyte sedimentation rate; ALB (g/L), albumin.

**Table 3:** Laboratory parameters between the three groups of patients.

Parameter	FSEC Group (*n* = 30)	MECT Group (*n* = 30)	CTFM Group (*n* = 30)	*p*
WBC	−0.49 (−1.14, 0.82)	0.65 (−0.54, 1.49)	0.08 (−1.07, 1.35)	0.22
NE	−0.05 (−0.58.1.10)	−0.24 (−0.91, 0.50)	0.33 (−0.72, 1.36)	0.53
RBC	−0.09 ± 0.50	−0.09 ± 0.54	−0.17 ± 0.56	0.80
HB	−1.50 ± 12.27	−2.48 ± 10.80	−4.10 ± 11.06	0.68
PLT	−29.5 (−61.00, −1.75)	−8.00 (−30.50, 33.00)	−13.50 (−38.75, 41.00)	0.05
CRP	3.24 ± 6.11	1.85 ± 3.55	13.41 ± 24.46	<0.05
ESR	5.00 (0.00, 12.25)	6.00 (1.50, 11.35)	4.50 (−0.25, 23.00)	0.98
ALB	−1.69 ± 3.64	−1.91 ± 3.09	−3.26 ± 5.49	0.32

Abbreviations: FSEC, Five-Flavor Sophora Flavescens Enteric-Coated Capsules; MECT, Mesalazine Enteric-Coated Tablet; CTFM, combination therapy of FSEC and MECT; *p*, *p*-value; WBC (×10^9^/L), white blood cell count; NE (×10^9^/L), neutrophil count; RBC (×10^12^/L), red blood cell count; HB (g/L), hemoglobin; PLT (×10^9^/L), platelet count; CRP (mg/L), C-reactive protein; ESR (mm/h), erythrocyte sedimentation rate; ALB (g/L), albumin.

**Table 4:** The core target of FSEC in the treatment of UC.

Gene Name	Full Name	Main Functions	Correlation with UC
ICAM1 [31]	Intercellular Adhesion Molecule-1	It mediates leukocyte adhesion and transmigration across the endothelium.	Alicaforsen, an antisense oligonucleotide targeting ICAM-1, has been evaluated in clinical trials for UC, although with limited efficacy as a systemic therapy.
CXCL2 [32]	C-X-C motif chemokine	A potent chemoattractant directing neutrophil migration to sites of inflammation.	Identified as a genetic risk marker in IBD transcriptome analysis, suggesting involvement in neutrophil-driven intestinal inflammation.
HMOX1 [33]	Heme Oxygenase 1	A cytoprotective enzyme that degrades heme into antioxidant products, exerting anti-inflammatory and anti-apoptotic effects.	Modulates intestinal inflammation by protecting colonic mucosa from oxidative injury; its pharmacological activation attenuates experimental colitis.
F3 [34,41]	Coagulation factor III (Tissue factor)	Activation of the extrinsic coagulation pathway may contribute to local microthrombus formation, exacerbating mucosal injury.	TF-dependent PAR-2 activation in colonic epithelial cells leads to local keratinocyte-derived chemokine production, triggering granulocyte influx, leading to inflammation and organ damage.
THBD [35,42]	Thrombomodulin	Glycomembrane protein and activator protein C system in vascular endothelial cells exert anticoagulant, anti-inflammatory, and endothelial protective effects.	It was significantly increased in patients with active IBD. It may play a regulatory role in fibrin deposition in inflammatory sites and/or in the progression of inflammatory processes.
PLAU [36]	Plasminogen activator, urokinase	Protein-coding genes convert plasminogen into plasmin. Involved in tumor cell invasion and metastasis.	PLAU is one of the potential therapeutic targets predicted by network pharmacology.
SLC6A4 [37,43]	Solute carrier family 6-member 4	It encodes serotonin transporter (SERT), regulates 5-hydroxytryptamine reuptake and affects the function of ‘brain-gut axis’.	5-HT inhibits intestinal inflammation through the 5-HT7R/RIPK1 pathway
MAOB [44]	Monoamine oxidase B	Metabolize monoamine neurotransmitters such as 5-hydroxytryptamine and dopamine	To control 5-HT degradation by regulating MAOB activity, attention should be paid to avoid excessive accumulation of 5-HT caused by MAOB inhibition.
DPP4 [38]	Dipeptidyl peptidase 4	A serine protease, widely involved in blood glucose regulation, immune response and cell signal transduction.	IBD potential biomarkers, its inhibitors significantly reduce colonic inflammation by inhibiting GLP-2 degradation. DPP4 gene knockout induces different outcomes in different species, indicating the complexity of DPP4 function.
CYP3A4 [39]	Cytochrome P450 3A4	It is an important member of the cytochrome P450 enzyme system, mainly involved in drug metabolism, and its expression is decreased in the inflammatory state.	Intestinal inflammation inhibits the expression of intestinal P450 and changes the pharmacokinetics of some drugs.
APOB [40]	Apolipoprotein B	The main structural proteins of low-density lipoprotein (LDL) and very low-density lipoprotein (VLDL) are responsible for lipid transport and metabolism.	Given that UC is often accompanied by dyslipidemia, impaired APOB function may disrupt intestinal lipid absorption or transport, thereby exacerbating inflammation.

## Data Availability

The original contributions presented in this study are included in the article/Supplementary Material; further inquiries can be directed to the corresponding authors.

## Supplementary Materials

The following supporting information can be downloaded at: https://www.mdpi.com/article/10.3390/biomedicines14061236/s1, Supplementary file S1: Table S1: Reagents, drugs, and key equipment; Table S2: Disease activity index score; Table S3: Histopathological score; Table S4: The primer sequence for qRT-PCR. Supplementary file S2: Table S5: The corresponding drug component names and drug gene targets of FSEC; Table S6: Differentially expressed genes in the GSE24287 dataset; Table S7: Top 15 genes identified by seven algorithms in cytoHubba; Table S8: GO enrichment analysis of the core genes; Table S9: KEGG pathway enrichment analysis of the core genes. Supplementary file S3: Table S10: Baseline characteristics of FSEC and MECT groups after 1:1 PSM; Table S11: Baseline characteristics of CTFM and matched monotherapy groups after 1:2 PSM; Table S12: Laboratory parameters pre- and post-treatment in the FSEC group; Table S13: Laboratory parameters pre- and post-treatment in the MECT group; Table S14: Laboratory parameters pre- and post-treatment in the CTFM group; Table S15: Detailed adverse events in the three groups of patients.
