# Supplementary material for: Targeting the AGE-RAGE/NF-κB Pathway: An Integrative Study Decoding the Anti-Colitis Effect of Five-Flavor Sophora Flavescens Enteric-Coated Capsule
Source: Biomedicines. 2026 May 29;14(6):1236. doi: 10.3390/biomedicines14061236 (PMC13296403; doi:10.3390/biomedicines14061236)
Supplement: Supplementary file 1 [file biomedicines-14-01236-s001.zip › biomedicines-4266878-supplementary/Supplementary file S1.pdf]

Supplementary Material S1

1.

Table S1 Reagents, drugs, and key equipment

Item/Product Name	Specification / Model / Dilution (if applicable)	Supplier / Manufacturer	Origin (Country)
Dextran Sulfate Sodium (DSS)	CD08040 (Molecular weight: 36,000-50,000Da)	MP Biomedicals, LLC	Santa Ana, CA，USA
Fecal Occult Blood Reagent	BA2020B	Zhuhai Beso Biotechnology Co., Ltd.	Zhuhai, China
Rabbit monoclonal anti-RAGE	1:500, 16346-1-AP (IHC)	Proteintech Group	Wuhan, China
Rabbit monoclonal anti-phospho-NF-κB-p65	1:100, CY6372 (IHC)	ABWAYS Technology	Shanghai, China
Rabbit monoclonal anti-NF-κB p65	1:50, CY2329 (IHC)	ABWAYS Technology	Shanghai, China
Rabbit monoclonal anti-RAGE	1:1000, ab216329 (Western Blot)	Abcam plc	Cambridge, UK
Rabbit monoclonal anti-phospho-NF-κB-p65	1:1000, #3033 (Western Blot)	Cell Signaling Technology	Danvers, MA, USA
Rabbit monoclonal anti-NF-κB p65	1:1000, #8242 (Western Blot)	Cell Signaling Technology	Danvers, MA, USA
Recombinant Anti-GAPDH antibody (HRP Conjugated)	1:3000, ZB15004-HRP-100	Servicebio Technology Co.,Ltd	Wuhan, China
HRP-conjugated goat anti-rabbit IgG	1:500, SA00001-2 (IHC)	Proteintech Group, Inc	Wuhan, China
HRP-conjugated goat anti-rabbit IgG	1:10000, SA00001-2 (Western Blot)	Proteintech Group, Inc	Wuhan, China
Mouse IL-1β ELISA Kit	SYP-M0026	UpingBio technology Co.,Ltd	Huangshi, China
Mouse TNF-α ELISA Kit	SYP-M0036	UpingBio technology Co.,Ltd	Huangshi, China

Mouse IL-6 ELISA Kit	SYP-M0031QX	UpingBio technology Co.,Ltd	Huangshi, China
Mouse AGEs ELISA Kit	SYP-M1947	UpingBio technology Co.,Ltd	Huangshi, China
SuperKine West Femto Maxi mum Sensitivity Substrate	BMU102-CN	Abbkine Scientific Co., Ltd	Wuhan, China
AG RNAex Rro Reagent	AG21102	Accurate Biotechnology (HUNAN） CO.,LTD	Changsha, China
Evo M-MLV Reverse Transcription Kit	AG11728	Accurate Biotechnology (HUNAN） CO.,LTD	Changsha, China
SYBR Green Premix Pro Taq HS qPCR Kit	AG11701	Accurate Biotechnology (HUNAN） CO.,LTD	Changsha, China
RAW264.7 cells	-	Procell Life Science & Technology Co., Ltd	Wuhan, China
MEM medium	PM50411	Procell Life Science & Technology Co., Ltd	Wuhan, China
Penicillin-Streptomycin solution	PB180120	Procell Life Science & Technology Co., Ltd	Wuhan, China
Fetal bovine serum (FBS)	16140071	Thermo Fisher Scientific, Inc.	Waltham, MA, USA
Nitric Oxide (NO) Detection Kit	S0021S	Beyotime Biotechnology, Inc.	Shanghai, China
Cell Counting Kit-8 (CCK-8)	BMU106-CN	Abbkine Scientific Co., Ltd	Wuhan, China

D-Ribose (D-Rib)	HY-W018772	MedChemExpress LLC	Monmouth Junction, NJ, USA
Lipopolysaccharide (LPS)	L8880	Solarbio Science & Technology Co., Ltd	Beijing, China
Five-Flavor Sophora Flavescens Enteric-Coated Capsule (FSEC)	National Drug Approval No. Z20150002	Beijing Zhonghui Pharmaceutical Co., Ltd	Beijing, China
Mesalazine Enteric-Coated Tablet (MECT)	Approval No. H20171358	Losan Pharma GmbH	Neuenburg, Germany
Microplate Reader	Infinite M200	Tecan US, Inc.	Morrisville, NC, USA
Gel Imaging System	GenoSens 2000	Clinx Scientific Instruments Co., Ltd.	Shanghai, China
Real-time PCR System and Thermal Cycler	7500	Applied Biosystems	Foster City, CA, USA
CO2 Incubator	Vios iDx 165	Thermo Fisher Scientific	Waltham, MA, USA
Inverted Microscope	CKX53	Olympus Corporation	Tokyo, Japan
ImageJ software	Version 1.53t	National Institutes of Health	Bethesda, MD, USA

2.

Table S2 Disease activity index score

Score	Body weight loss (%)	Stool consistency	Fecal occult blood
0	0	Normal	Negative
1	1-5	Soft but formed	Faint blue
2	5-10	Loose	Blue
3	10-15	Watery	Dark blue
4	>15	Severe diarrhea	Gross bleeding

Fecal occult blood was determined using a commercial fecal occult blood test kit (BA2020B, Baso Diagnostics Inc., Zhuhai, China) according to the manufacturer's instructions.

Briefly, 10–50 mg of fecal specimen was applied to the test card, followed by sequential addition of Developer A and Developer B. Results were read within 2 minutes after adding Developer B and

scored semi-quantitatively as: negative (−), faint purple-red within 1–2 min (+), purple-red within 1 min (++), deep purple-blue within 10 s (+++), or immediate deep purple-blue (++++).

3.

Table S3 Histopathological score

Score	Inflammatory cell infiltration	Tissue damage
0	No inflammatory cell infiltration	No mucosal damage
1	Increased inflammatory cells in the lamina propria	Dispersed lesions in epithelium
2	Submucosal infiltration of inflammatory cells	Erosion or focal ulcers
3	Transmural infiltration of inflammatory cells	Severe mucosal damage with widespread ulcers extending through the intestinal wall

4.

Table S4 The primer sequence for qRT-PCR

Genes	Sequence of premers (5'-3')
TNF-a (mouse)	F: ACTCCAGGCGGTGCCTATGT; R: GTGAGGGTCTGGGCCATAGAA
IL-1β (mouse)	F: AATGAAAGACGGCACACCCA; R: ACTCCACTTTGCTCTTGACTTCT
IL-6 (mouse)	F:CCACTTCACAAGTCGGAGGCTTA; R: TGCAAGTGCATCATCGTTGTTC
RAGE (mouse)	F: AGGAGGTCAAGTCCAACTACC; R: TAGCTTCCCTCAGACACACAT
GAPDH (mouse)	F: TGTGTCCGTCGTGGATCTGA; R: TTGCTGTTGAAGTCGCAGGAG

5. Statistical Analysis

All statistical analyses were performed using SPSS software (version 26.0) and R software (version 4.2.1). Data are presented as mean ± standard deviation (SD) for normally distributed variables, median (interquartile range, IQR) for non-normally distributed variables, and number (percentage) for categorical variables. Prior to group comparisons, the normality of data distribution for all continuous variables was assessed using the Shapiro-Wilk test, and the homogeneity of variances was evaluated using Levene's test.

5.1 Unadjusted Analyses

For comparisons between multiple groups, a one-way analysis of variance (ANOVA) was applied for data meeting parametric assumptions, followed by Tukey's post hoc test for pairwise comparisons. For data that did not satisfy the assumptions of normality or homogeneity of variances, the Kruskal-Wallis H test was employed, followed by Dunn's post hoc test with Bonferroni correction for multiple comparisons. For comparisons within the same group, a paired-sample *t*-test or the Wilcoxon signed-rank test was used. For categorical clinical data, the chi-square test or Fisher's exact test was used as appropriate. To control for type I error due to multiple comparisons, all pairwise comparisons of clinical outcomes (clinical remission rate and mucosal healing rate) were adjusted using the Bonferroni method, with a corrected significance level of $\alpha' = 0.05/3 = 0.0167$ for three-group comparisons.

5.2. Propensity Score Matching (PSM)

we performed PSM using the MatchIt package in R. Two separate PSM analyses were conducted:

Comparison A: FSEC monotherapy vs. MECT monotherapy

A 1:1 nearest-neighbor matching algorithm was used with a caliper width of 0.2 standard deviations of the logit of the propensity score. The propensity score was esti-mated using a logistic regression model that included the following clinically relevant covariates: age, sex, duration of UC, BMI, smoking history, extraintestinal manifestations, disease extent, clinical course, and baseline disease severity. Standardized mean differences (SMD) were calculated to assess the balance of covariates between groups before and after matching, with an SMD < 0.1 considered indicative of good balance.

Comparison B: CTFM combination therapy vs. pooled monotherapy

The FSEC and MECT groups were combined to create a pooled monotherapy cohort ($n = 60$). A 1:2 nearest-neighbor matching algorithm was used with a caliper width of 0.2 standard deviations of the logit of the propensity score. The same set of covariates as in Comparison A was used to estimate the propensity score. After matching, we compared clinical remission rates, mucosal healing rates, and changes in laboratory parameters between the CTFM group and the matched monotherapy group.

5.3. Interobserver Agreement Analysis

Interobserver agreement for mucosal healing assessment was calculated using Co-hen's kappa coefficient. A kappa value of 0.81-1.00 was considered excellent agreement, 0.61-0.80 substantial agreement, 0.41-0.60 moderate agreement, 0.21-0.40 fair agreement, and 0.00-0.20 slight agreement.

5.4 Multivariate logistic Regression Analysis

The dependent variables were clinical remission and mucosal healing. The independent variables included treatment group (FSEC vs. MECT; CTFM vs. pooled monotherapy) and the same baseline covariates used in the PSM analysis. Results are presented as odds ratios (OR) with 95% confidence intervals (CI).

5.5. Retrospective Sample Size and Power Analysis

Retrospective sample size and power analysis were performed using GPower software (version 3.1.9.7) to assess whether the study had sufficient statistical power to detect meaningful differences in the primary endpoint (clinical remission rate). We used a chi-square test for two independent proportions with a two-sided α level of 0.05 and calculated the statistical power to detect a 15% absolute difference in clinical remission rates between the FSEC group and MECT group.

A p-value of < 0.05 was considered statistically significant, except where otherwise specified for multiple comparison corrections.
