# Supplementary material for: Targeting the AGE-RAGE/NF-κB Pathway: An Integrative Study Decoding the Anti-Colitis Effect of Five-Flavor Sophora Flavescens Enteric-Coated Capsule
Source: Biomedicines. 2026 May 29;14(6):1236. doi: 10.3390/biomedicines14061236 (PMC13296403; doi:10.3390/biomedicines14061236)
Supplement: Supplementary file 1 [file biomedicines-14-01236-s001.zip › biomedicines-4266878-supplementary/Supplementary file S3.pdf]

Supplementary Material S3

1

1.1 Baseline Characteristics After PSM

Table S10 Baseline characteristics of FSEC and MECT groups after 1:1 PSM.

Variables	FSEC group (*n* = 30)	MECT group (*n* = 30)	SMD
Age (years)	47.23±15.55	48.17±15.89	0.06
Gender-male (*n*)	17	16	0.07
Duration of UC（month）	24.00（9.50, 44.50）	23.50（8.75, 43.25）	0.03
BMI	21.70（19.58, 22.75）	21.78（19.65, 22.82）	0.02
Non-smoker (*n*)	23	23	0.00
No EIMs (*n*)	26	26	0.00
Disease extent（*n*）			
E1	5	5	0.00
E2	10	10	0.00
E3	15	15	0.00
Clinical course(*n*)			
First episode	6	6	0.00
Chronic continuous	24	24	0.00
Severity (*n*）			
Mild	14	14	0.00
Moderate	16	16	0.00

Abbreviations: FSEC, Five-Flavor Sophora Flavescens Enteric-Coated Capsules; MECT, Mesalazine Enteric-Coated Tablet; BMI, body mass index; EIMs, extraintestinal manifestations.

Table S11 Baseline characteristics of CTFM and matched monotherapy groups after 1:2 PSM.

Variables	CTFM group (*n* = 30)	Matched monotherapy group (*n* = 60)	SMD
Age (years)	54.07±16.96	53.62±16.78	0.03
Gender-male (*n*)	20	39	0.04
Duration of UC (month)	27.00（2.00, 93.75）	26.50（1.75, 92.50）	0.02
BMI	21.64（20.40, 23.68）	21.67（20.43, 23.71）	0.01

Variables	CTFM group (n = 30)	Matched monotherapy group (n = 60)	SMD
Non-smoker (n)	25	50	0.00
No EIMs(n)	25	50	0.00
Disease extent (n)			
E1	2	4	0.00
E2	13	26	0.00
E3	15	30	0.00
Clinical course(n)			
First episode	8	16	0.00
Chronic continuous	22	44	0.00
Severity (n)			
Mild	8	16	0.00
Moderate	21	42	0.00

Abbreviations: CTFM, combination therapy of FSEC and MECT; BMI, body mass index; EIMs, extraintestinal manifestations.

1.2 Comparison of Laboratory Parameters Related to UC at Baseline and After Treatment

Table S12 Laboratory parameters pre- and post-treatment in the FSEC group

Parameter	Pre	Post	t/z	p
WBC	6.17±1.76	6.53±2.77	-0.697	0.49
NE	3.77±1.26	3.86±2.11	-0.238	0.81
RBC	4.36±0.45	4.45±0.38	-0.97	0.34
HB	127.13±15.73	128.63±10.94	-0.669	0.51
PLT	250.77±54.73	283.07±54.58	-3.12	＜0.05
CRP	8.14±6.91	4.90±5.15	2.90	＜0.05
ESR	17.07±16.61	8.60±5.74	3.50	＜0.05
ALB	39.31±3.38	41.00±2.16	-2.54	＜0.05

Abbreviations: FSEC, Five-Flavor Sophora Flavescens Enteric-Coated Capsules; Pre, Pre-treatment; Post, Post-treatment; p, p-value; WBC($\times 10^9$/L), white blood cell count; NE($\times 10^9$/L), neutrophil count; RBC($\times 10^{12}$/L), red blood cell count; HB(g/L), hemoglobin; PLT($\times 10^9$/L), platelet count; CRP(mg/L), C-reactive protein; ESR(mm/h), erythrocyte sedimentation rate; ALB(g/L), albumin.

Table S13 Laboratory parameters pre- and post-treatment in the MECT group

Parameter	Pre	Post	t/z	*p*
WBC	6.46±2.01	5.77±1.35	2.06	＜0.05
NE	3.62±1.46	3.60±0.96	0.06	0.95
RBC	4.47±0.59	4.57±0.53	-1.03	0.31
HB	132.50±17.99	135.43±15.15	-1.47	0.15
PLT	241.86±61.81	248.62±58.73	-0.70	0.94
CRP	5.63±4.78	3.77±3.26	2.94	＜0.05
ESR	15.62±11.79	9.14±7.94	4.55	＜0.05
ALB	39.73±3.48	41.65±2.64	-3.59	＜0.05

Abbreviations: MECT, Mesalazine Enteric-Coated Tablet; Pre, Pre-treatment; Post, Post-treatment; *p*, *p*-value; WBC($\times 10^9$/L), white blood cell count; NE($\times 10^9$/L), neutrophil count; RBC($\times 10^{12}$/L), red blood cell count; HB(g/L), hemoglobin; PLT($\times 10^9$/L), platelet count; CRP(mg/L), C-reactive protein; ESR(mm/h), erythrocyte sedimentation rate; ALB(g/L), albumin.

Table S14 Laboratory parameters pre- and post-treatment in the CTFM group

Parameter	Pre	Post	t/z	*p*
WBC	7.02±2.65	6.51±1.84	1.09	0.28
NE	4.45±2.00	4.13±1.75	1.11	0.27
RBC	4.25±0.46	4.43±0.63	-1.69	0.10
HB	122.00±16.82	126.10±10.04	-2.03	0.052
PLT	265.70±73.90	270.03±67.18	-0.38	0.71
CRP	21.89±34.68	8.47±12.58	3.00	＜0.05
ESR	24.60±24.85	15.76±18.88	2.52	＜0.05
ALB	36.62±5.74	39.89±3.55	-3.26	＜0.05

Abbreviations: CTFM, combination therapy of FSEC and MECT; Pre, Pre-treatment; Post, Post-treatment; *p*, *p*-value; WBC($\times 10^9$/L), white blood cell count; NE($\times 10^9$/L), neutrophil count; RBC($\times 10^{12}$/L), red blood cell count; HB(g/L), hemoglobin; PLT($\times 10^9$/L), platelet count; CRP(mg/L), C-reactive protein; ESR(mm/h), erythrocyte sedimentation rate; ALB(g/L), albumin.

1.3 Adverse Events

Table S15 Detailed adverse events in the three groups of patients.

Adverse Event	FSEC group (n = 30)	MECT group (n = 30)	CTFM group (n = 30)	Total (n = 90)
Gastrointestinal disorders				
Nausea	1 (3.3%)	2 (6.7%)	1 (3.3%)	4 (4.4%)
Diarrhea	0 (0.0%)	0 (0.0%)	0 (0.0%)	0 (0.0%)
Abdominal pain	0 (0.0%)	0 (0.0%)	0 (0.0%)	0 (0.0%)
Skin and subcutaneous tissue disorders				
Rash	0 (0.0%)	1 (3.3%)	1 (3.3%)	2 (2.2%)
Pruritus	0 (0.0%)	0 (0.0%)	0 (0.0%)	0 (0.0%)
Nervous system disorders				
Dizziness	0 (0.0%)	1 (3.3%)	0 (0.0%)	1 (1.1%)
Hepatobiliary disorders				
ALT elevation	1 (3.3%)	0 (0.0%)	0 (0.0%)	1 (1.1%)
Total adverse events	2 (6.7%)	4 (13.3%)	2 (6.7%)	8 (8.9%)

Abbreviations: FSEC, Five-Flavor Sophora Flavescens Enteric-Coated Capsules; MECT, Mesalazine Enteric-Coated Tablet; CTFM, combination therapy of FSEC and MECT; ALT, alanine aminotransferase.
